# Long-Term Treatment Outcomes of Patients Infected With Hepatitis C Virus: A Systematic Review and Meta-analysis of the Survival Benefit of Achieving a Sustained Virological Response

**DOI:** 10.1093/cid/civ396

**Published:** 2015-05-17

**Authors:** Bryony Simmons, Jawaad Saleem, Katherine Heath, Graham S. Cooke, Andrew Hill

**Affiliations:** 1 Division of Medicine, Imperial College London; 2 Pharmacology and Therapeutics, Liverpool University, United Kingdom

**Keywords:** hepatitis C, sustained virologic response, mortality, survival

## Abstract

***Background.*** Achievement of a sustained virologic response (SVR) after treatment for Hepatitis C infection is associated with improved outcomes. This meta-analysis aimed to determine the impact of SVR on long-term mortality risk compared with nonresponders in a range of populations.

***Methods.*** An electronic search identified all studies assessing all-cause mortality in SVR and non-SVR patients. Eligible articles were stratified into general, cirrhotic, and populations coinfected with human immunodeficiency virus. The adjusted hazard ratio (95% confidence interval [CI]) for mortality in patients achieving SVR vs non-SVR, and pooled estimates for the 5-year mortality in each group were calculated.

***Results.*** 31 studies (n = 33 360) were identified as suitable for inclusion. Median follow-up time was 5.4 years (interquartile range, 4.9–7.5) across all studies. The adjusted hazard ratio of mortality for patients achieving SVR vs non-SVR was 0.50 (95% CI, .37–.67) in the general population, 0.26 (95% CI, .18–.74) in the cirrhotic group, and 0.21 (.10–.45) in the coinfected group. The pooled 5-year mortality rates were significantly lower for patients achieving SVR compared with non-SVR in all 3 populations.

***Conclusions.*** The results suggest that there is a significant survival benefit of achieving an SVR compared with unsuccessful treatment in a range of populations infected with hepatitis C virus.

## Abstract (precis)

The results of this meta-analysis suggest that there is a significant survival benefit of achieving an sustained virologic response compared with unsuccessful treatment in the general hepatitis C virus-infected population. This benefit is held in patients with cirrhosis and those coinfected with human immunodeficiency virus.

Hepatitis C virus (HCV) is a significant public health concern with an estimated 185 million people infected worldwide [1]. HCV progression can lead to the development of liver cirrhosis and hepatocellular carcinoma and results in the deaths of over 700 000 people every year [2]. Combined, viral hepatitis kills more people per year than malaria or tuberculosis but has commanded far less attention and access to care and treatment is limited [2, 3].

Traditionally, treatment for HCV has composed of dual-therapy with pegylated-interferon and ribavirin. Dual-therapy is associated with poor sustained virological response (SVR) rates, the surrogate marker for cure defined as undetectable HCV RNA 24 weeks following completion of therapy. A robust treatment pipeline has seen the recent approval of highly efficacious interferon-free regimens with a number of other therapy combinations likely to be approved over the next 2 years. These novel treatment regimens will have the potential to transform the treatment landscape [4, 5]. Promisingly, the high response rate is matched in populations typically considered difficult-to-treat, such as those with advanced fibrosis or coinfection with human immunodeficiency virus (HIV) [6, 7].

Relative to nonresponders or to those untreated, the attainment of an SVR has repeatedly been associated with improved patient outcomes, irrespective of the path to SVR. These include reduced incidence of liver decompensation, hepatocellular carcinoma, and death [8–10]. Evidence suggests that an SVR does not only prevent the progression of liver disease but is associated with histologic improvements with some studies even reporting the complete resolution of fibrosis after SVR [10, 11]. Moreover, SVR-achievement has been associated with a reduction in extra-hepatic events and a reduction in mortality independent of liver disease [10, 12–16].

Despite the evidence for improved prognosis with SVR, there are some contradictory data suggesting that SVR-achievement does not provide a significant clinical benefit [9, 17, 18]. A number of studies have shown that the risk of progression is not eliminated with viral eradication, with some patients experiencing decompensation or developing hepatocellular carcinoma despite achieving an SVR [10, 11, 19, 20]. Furthermore, some evidence suggests that the improved prognosis associated with SVR may be diminished in certain patient groups such as those with decompensation or HIV coinfection [12, 21]. There is a need for definitive evidence evaluating the clinical benefit of achieving an SVR in a range of populations, especially given the high cost of interferon-free regimens [4].

The aim of this study was to systematically review the current literature concerning the survival benefits of achieving SVR through treatment vs the outcomes in nonresponders and relapsers (non-SVR). All-cause mortality was chosen as the endpoint as it is definitive with clear interpretation. Further, given the extra-hepatic benefits of SVR, all-cause mortality may be clinically more relevant than liver-related mortality.

## METHODS

We evaluated the mortality rates of patients after treatment for chronic HCV to determine whether, and to what extent, SVR is a prognostic factor for subsequent all-cause mortality.

### Search Strategy and Selection Criteria

Studies for inclusion in the review were identified through an electronic search of 2 biomedical literature databases. The databases PubMed and EMBASE were searched for articles published between 1990 and November 2014 using a sensitive search string with keywords including HCV, SVR, and mortality. No language or geographical restrictions were applied. The search was supplemented by a thorough review of the reference lists of all articles fulfilling eligibility criteria and a search of the proceedings from relevant conferences. Conference proceedings were searched for any relevant articles from 2000 to 2014 and included the American Association for the Study of Liver Diseases, European Association for the Study of the Liver, Asian Pacific Association for the Study of the Liver, Conference on Retroviruses and Opportunistic Infections, and the International AIDS Conference. Two independent authors (B. S. and J. S.) reviewed the process, ensuring the papers met the inclusion criteria and independently extracted the data for review. Any disagreements were resolved by consensus or arbitration by a third reviewer.

Any retrospective or prospective observational study assessing prognosis of HCV with treatment and any randomized controlled trial assessing the impact of SVR vs non-SVR was eligible for inclusion in the study. Participants had to be adults (>18 years old) chronically infected with HCV of any genotype and were treated with any antiviral regimen for the recommended duration. SVR-achievement was defined as undetectable viremia 24 weeks after completion of antiviral therapy (SVR24); all patients with a detectable viral load at the SVR24 time-point, inclusive of those with an end-of-treatment response, were considered nonresponders and were included in the non-SVR arm. Only trials with a post-therapy follow-up of longer than 1 year were included, and only patients alive at the SVR24 time-point were included in the analyses. Studies were to evaluate all deaths irrespective of cause (all-cause mortality); studies restricted to liver-related mortality were excluded from the current review.

The eligible articles were stratified into 3 patient populations as follows: (1) General: studies of monoinfected patients at all disease stages; (2) Cirrhotic: studies of monoinfected patients with advanced fibrosis or cirrhosis; (3) HIV/HCV coinfected: all studies of HIV/HCV coinfected patients, regardless of baseline fibrosis status. The following details were extracted from all studies: study location, study type, baseline characteristics, number of patients treated and number achieving SVR, number of deaths in each arm, duration of patient follow-up, and where possible, the hazard ratios (HRs) of mortality. Where data were missing, authors were contacted to retrieve the information; studies with missing follow-up time or other essential raw outcome data were excluded if data were not retrievable. In the case of duplicate studies, the report covering the longest time period with the largest population was used.

### Quality Assessment

Study quality was evaluated using the Quality in Prognosis Studies (QUIPS) tool, which considers the following 6 domains of bias: participation, attrition, prognostic factor measurement (SVR-attainment), outcome measurement (all-cause mortality), confounding, and analysis and reporting [22]. For each study, each domain was considered as having a high, moderate, or low risk of bias based on a list of prompting study aspects. A bias risk for the analysis domain was only determined in those studies reporting adjusted results.

### Data Analysis

For each of the 3 populations, the 5-year mortality rate after treatment was calculated for the SVR and non-SVR arms. The log-transformed incidence rate (IR) and corresponding standard error for each study was calculated using the number of events (deaths) and person-years of follow-up (PYFU). A Poisson distribution was assumed for calculation of the standard error and results were pooled using a random-effects model according to the methods of DerSimonian and Laird [23]. The results were converted to 5-year estimates and presented along with the corresponding 95% confidence interval (CI). A 5-year horizon was deemed most appropriate as the follow-up period in the majority of studies did not exceed this time-point (median follow-up 5.4 years [interquartile range {IQR}, 4.9–7.5]). Plots of IR against follow-up time were visually inspected to test the assumption that the mortality rate was constant over this timespan.

A comparison of the risk of death in the SVR group vs the non-SVR group was conducted by pooling the HRs for mortality. The HRs reported in each study were calculated using Cox proportional hazards models, and both the unadjusted and adjusted HRs were extracted along with the corresponding variances. As above, pooled estimates for the adjusted HRs were computed using a random-effects model. Where necessary, variance was calculated according to the methods of Parmar et al [24]. Heterogeneity across studies was quantitatively assessed using the I^2^ statistic in accordance with the Cochrane Handbook [25]. All analyses were conducted using Review Manager (RevMan version 5.3; Cochrane Collaboration) and Stata (STATA 12; StataCorp LP).

### Publication Bias

The existence of publication bias was assessed using funnel plots. Statistical tests for asymmetry are low powered, and as such, given the small number of studies anticipated per group, funnel plots were interpreted by visual inspection.

## RESULTS

### Search Results

The search strategy initially yielded 4877 articles, of which 4746 were found to be irrelevant and were excluded. A further 11 potential studies were identified through the reference list review and the search of conference proceedings. Of the final 142 articles, 31 (n = 33 360) fitted the criteria for inclusion. The main reasons for exclusion included absence of mortality data, unclear recording of essential outcomes, including follow-up time, number with SVR, and number of deaths, and duplication of studies. Of the final 31 studies, 17 were in patients at any stage of liver fibrosis (general studies; n = 28 398), 9 were in cirrhotic patients (n = 2604), and the remaining 5 studies were of HIV/HCV coinfected patients (n = 2358). The median of the median follow-up time was 5.2 years (IQR, 4.3–7.8) in the general studies, 6.8 years (IQR, 5.8–7.9) in the cirrhotic studies, and 5.0 years (IQR, 4.6–5.2) in the coinfected studies. The majority of studies were carried out in European, Asian, or North American settings. Participants were predominantly male, infected with HCV genotype 1, and between the ages of 40 and 50 at baseline. All participants were treated with interferon or pegylated-interferon, either as monotherapy or in combination with ribavirin. Study characteristics are shown in Table 1.

**Table 1.** Details of Included Study Populations

Study	Country (Analysis Type)	Treatment Regimen	Follow-up, Years	No. Treated With FU (% With SVR)	Mean Age (SD)^a^	Male, %	Fibrosis Staging	Genotype
**General cohorts**								
Giannini 2001 [26]	Italy (prospective)	IFN-α	3.0	36 (42)	44 ± 11	78	7.3 ± 3.6^b^	GT1b 33%; non-GT1b 67%
Yoshida 2002 [27]	Japan (retrospective)	IFN-α or IFN-β	5.4	2430 (34)	50 ± 11	63	70% ≥F2; 9% F4	NR
Imazeki 2003 [28]	Japan (retrospective)	IFN-α or IFN-β	8.3	355 (33)	49 ± 12	64	44% ≥F2; 13% F4	GT1 74%; non-GT1 26%
Veldt 2004 [29]	Europe (retrospective)	IFN or IFN-α	4.9	336 (85)	42 (17–72)	58	8% cirrhotic	GT1 40%; non-GT1 60%
Kasahara 2004 [30]	Japan (retrospective)	IFN monotherapy	5.8	2698 (28)	53 (20–76)	64	71% ≥F2; 5% F4	NR
Coverdale 2004 [19]	Australia (prospective)	IFN-α	8.0	343 (15)	37 (32–49)	67	19% cirrhotic	GT1 38%; non-GT1 62%
Yu 2006 [31]	Taiwan (retrospective-prospective)	IFN-α ± RBV	5.2	1057 (68)	47 ± 12	60	16% cirrhotic	GT1 46%; non-GT1 54%
Arase 2007 [32]	Japan (retrospective)	IFN-α or IFN-β ± RBV	7.5	500 (28)	64 ± 3	50	52% ≥F2; 14% F4	GT1b 60%; non-GT1b 40%
Backus 2011 [14]	United States Vets (retrospective)	Peg-IFN + RBV	3.7	16 864 (44)	52 ± 6	96	13% cirrhotic	GT1 72%; non-GT1 28%
Innes 2011 [33]	Scotland (retrospective)	IFN or Peg-IFN ± RBV	5.3	1215 (46)	42 ± 10	69	14% cirrhotic	GT1 36%; non-GT1 55%
Reimer 2011 [34]	Germany (retrospective)	Peg-IFN + RBV	3.0	508 (56)	50 ± 13	58	NR	GT1 57%; non-GT1 43%
Di Martino 2011 [35]	France (prospective)	IFN or Peg-IFN ± RBV	4.9	184 (32)	42 ± 13	67	70% ≥F2; 11% cirrhotic	GT1 57%; non-GT1 43%
Maruoka 2012 [36]	Japan (retrospective)	IFN-α or IFN-β ± RBV	10.4	577 (38)	50 ± 12	64	47% ≥F2; 10% F4	GT1 31%; GT2 69%
Cozen 2013 [37]	United States (retrospective)	IFN-α ± RBV	10.0	140 (49)	60 ± 7	99	59% ≥F2; 11% F4	GT1 66%; non-GT1 34%
Rutter 2013 [38]	Austria (NR)	IFN or Peg-IFN ± RBV	5.0	454 (73)	50 ± 12	62	38% F3/F4	GT1 66%; non-GT1 34%
Singal 2013 [39]	United States (retrospective)	Peg-IFN + RBV	5.2	217 (38)	48 (43–54)	51	17% cirrhotic	GT1 69%; non-GT1 31%
Dieperink 2014 [13]	United States Vets (retrospective)	IFN, Peg-IFN or CIFN ± RBV	7.5	536 (41)	51 ± 6	98	82% ≥F2; 27% F4	GT1 70%; non-GT1 30%
**Overall (17 studies)**			**5**.**2 (IQR 4**.**3–7**.**8)**^c^	**28 451 (42)**	**51**	**83**	**67% ≥F2; 12% F4**	**GT1 66%**
**Cirrhotic cohorts**								
Kumar 2005 [40]	India (prospective)	IFN-α ± RBV	1.6	25 (32)	52 ± 14	80	80% F4; 20% DC	GT1 31%; GT3 62%
Braks 2007 [41]	France (retrospective)	IFN-α or Peg-IFN ± RBV	7.6	113 (33)	54 ± 11	61	100% F4	GT1 61%; non-GT1 39%
Bruno 2007 [42]	Italy (retrospective)	IFN monotherapy	8.0	893 (14)	55 ± 9	63	100% F4	GT1 72%; non-GT1 28%
Mallet 2008 [43]	France (retrospective)	IFN-α or Peg-IFN ± RBV	9.8	96 (41)	45 (36–56)	60	100% F4	GT1 53%; non-GT1 47%
Morgan 2010 [20]	United States (prospective)	Peg-IFN + RBV	6.8	526 (27)	49 ± 8	72	100% ≥F3; 35% F4 (no DC)	GT1 87%; non-GT1 13%
Iacobellis 2011 [21]	Italy (prospective)	Peg-IFN + RBV	4.2	75 (32)	61 ± 9	63	100% DC	GT1 57%; non-GT1 43%
Van der Meer 2012 [44]	Europe and Canada (retrospective)	IFN, Peg-IFN or CIFN ± RBV	8.4	530 (36)	48 (42–56)	70	100% ≥F3; 54% F4 (no DC)	GT1 68%; non-GT1 32%
Aleman 2013 [45]	Sweden (prospective)	Peg-IFN + RBV	5.3	303 (36)	51 ± 9	68	100% F4	GT1 47%; non-GT1 53%
Kutala 2014 [46]	France (retrospective)	IFN-α or Peg-IFN ± RBV	5.9	325 (32)	49 (43–57)	68	100% ≥F3; 51% F4	GT1 55%; non-GT1 45%
**Overall (9 studies)**			**6**.**8 (IQR 5**.**8–7**.**9)**^c^	**2,886 (27)**	**51**	**67**	**100% ≥F3; 74% F4orDC**	**GT1 68%**
**HIV coinfected cohorts**								
Limketkai 2012 [47]	United States (prospective)	IFN-α or Peg-IFN + RBV	5.2	212 (17)	46 (41–50)	66	42% ≥F2	GT1 91%; non-GT1 9%
Berenguer 2012 [12]	Spain (retrospective-prospective)	IFN-α or Peg-IFN + RBV	5.0	1599 (39)	40 (37–43)	75	39% F3/F4	GT1 49%; non-GT1 51%
P-Gonzalez 2013 [48]	Brazil (retrospective)	Peg-IFN + RBV	2.0	42 (33)	44 (29–67)	78	48% F4	GT1 54%; non-GT1 46%
Mira 2013 [49]	Spain (prospective)	Peg-IFN + RBV	4.6	166 (26)	43 (39–48)	86	100% F4	GT1 58%; non-GT1 42%
Labarga 2014 [50]	Spain (retrospective)	Peg-IFN + RBV	6.1	339 (41)	41	78	40% F3/F4	GT1or4 73%
**Overall (5 studies)**			**5**.**0 (IQR 4**.**6–5**.**2)**^c^	**2,358 (36)**	**41**	**75**	**42% ≥F3**	**GT1 57%**

Abbreviations: CIFN, consensus interferon; DC, decompensated cirrhosis; FU, follow-up; GT, genotype; HIV, human immunodeficiency virus; IFN, interferon; IQR, interquartile range; NR, not reported; Peg-IFN, pegylated interferon; RBV, ribavirin; SD, standard deviation; SVR, sustained virologic response.

^a^ Median (IQR) reported when unavailable.

^b^ Mean hepatitc activity index score (SD).

^c^ Median of median follow-up times and IQR.

### Quality Assessment

Of the 31 included studies, 5.7% of the domains, that is, inclusion, attrition, prognostic factor measurement, outcome measurement, confounding, and analysis and reporting as assessed with the QUIPS tool, showed a high risk of bias, 26.1% showed a moderate risk, and 68.2% showed a low risk of bias (Supplementary Appendix 1). Twenty-three studies showed a moderate-to-high risk of bias in 1 or 2 domains; 6 showed a moderate-to-high risk of bias in 3 or 4 domains. Risks of bias were highest in the domain of prognostic factor measurement (high in 8/31 [25.8%] and moderate in 14/31 [45.2%]), due to follow-up not originating at the SVR time-point. In these studies, follow-up was often measured from initiation of treatment, and in some cases from biopsy that was conducted up to 1 year prior to treatment.

### Data Synthesis

#### Estimates of the 5-year Risk of Mortality

In the general population, 502 of 12 140 (54 651 PYFU) patients achieving an SVR died during follow-up equating to a pooled IR of 0.4/100PY (95% CI, .2–.7). In comparison, 1708 out of 16 258 (77 130 PYFU) non-SVR patients died (IR = 1.6/100PY, 95% CI, 1.2–2.3).

In the cirrhotic studies 45 of 778 (5352 PYFU) SVR patients died during follow-up (IR = 1.0/100PY, 95% CI, .7–1.5) vs 404 of 2108 (15 836 PYFU) non-SVR patients (IR = 3.4/100PY, 95% CI, 2.4–4.8). Finally, in the HIV coinfected population 11 of 857 (4333 PYFU) SVR patients (IR = 0.3/100PY, 95% CI, .1–.6) and 161 of 1501 (7683 PYFU) non-SVR patients died during follow-up (IR = 2.4/100PY, 95% CI, 1.3–4.2). Visual observation of the plots of IR against follow-up time showed no association between the length of follow-up and the risk of mortality in either the SVR or non-SVR groups in all 3 populations; it was thus deemed appropriate to determine the 5-year mortality rates from these data.

**Figure 1.**
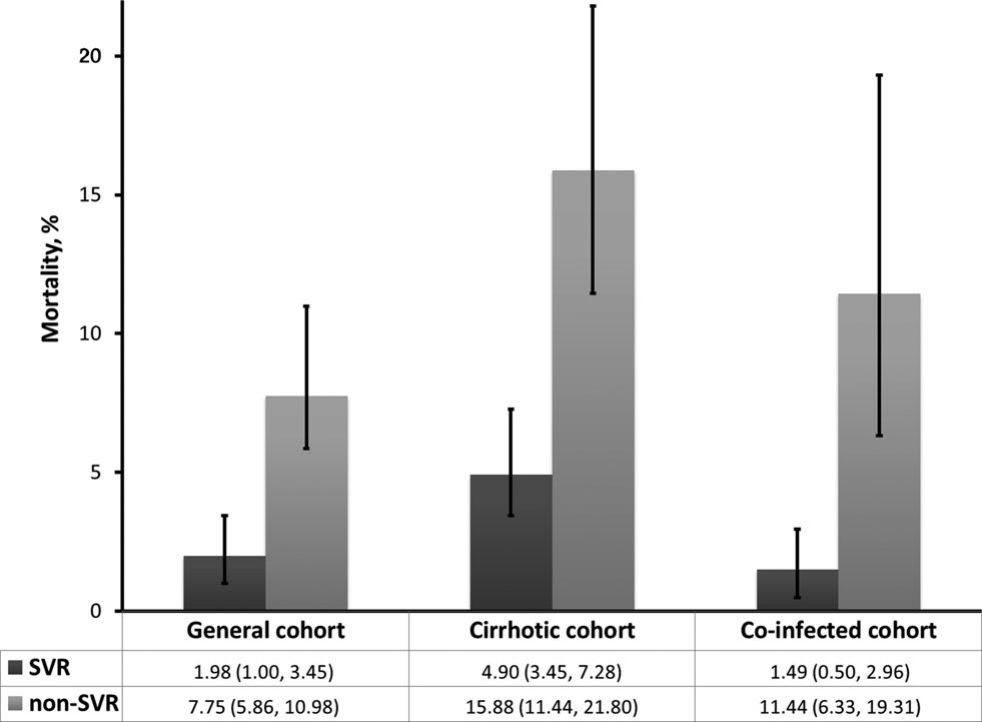
Five-year mortality rates (95% confidence interval) for sustained virologic response (SVR) vs non-SVR groups for each cohort.

As shown in Figure 1, the estimated 5-year mortality rate was significantly lower for patients achieving SVR compared with nonresponders for all 3 patient populations. The difference in mortality rate between SVR and non-SVR was most pronounced in the cirrhotic and coinfected populations.

#### Pooled Estimates of Hazard Ratios

Of the 31 studies included, 21 reported HRs for mortality adjusted for potential covariates that may have had an impact on the results. As shown in Table 2, the endpoint analyzed differed between studies. The majority of studies analyzed the rate of all-cause mortality, either alone (n = 12) or including liver-transplantation as a surrogate for mortality (n = 3). Of the remaining 6 studies, 5 evaluated liver-related deaths, and the last study evaluated non-liver related deaths. Furthermore, a number of studies compared mortality risk after SVR with the risk in untreated patients, in contrast with non-SVR (n = 7, all general studies). Most studies conducted a comprehensive analysis, adjusting for a variety of factors that may have impacted results, including age, gender, fibrosis stage, genotype, alcohol use, and comorbidities (Table 2).

**Table 2.** Univariate and Multivariate Hazard Ratios for Patients Achieving Sustained Virologic Response (SVR) vs Non-SVR for Each Cohort

Study, Year	Univariate	Multivariate	Covariates Adjusted for	Comparator for HR	Endpoint Analyzed
Mixed cohorts					
Yoshida 2002	NR	HR = **0.15 (.06–.34)**	Age, gender	Untreated	All-cause mortality
Imazeki 2003	HR = **0.21 (.07–.63)**	HR = **0.22 (.07–.71)**	Age, gender, BMI, fibrosis stage, treatment, AST, ALT, albumin, platelets, genotype, HCV core protein, alcohol consumption, duration of disease, diabetes, hypertension, fatty liver, chronic pulmonary disease	Untreated	All-cause mortality
Coverdale 2004	HR = **0.24 (.13–.43)**	NS (*P* = .2)	Age, duration of infection, place of birth, mode of transmission, genotype, fibrosis score, albumin, bilirubin, prothrombin time	Untreated	Liver-events & mortality
Kasahara 2004	NR	HR = **0.14 (.06–.35)**	Age, gender, stage of liver fibrosis, period at liver biopsy	Untreated	All-cause mortality
Yu 2006	NR	HR = **0.37 (.14–.99)**	Age, gender, genotype, treatment type, cirrhosis, ALT	Untreated	All-cause mortality
Arase 2007	HR = **0.37 (.17–.83)**	HR = **0.39 (.16–.93)**	Age, sex, liver histology, HCV VL, genotype, AST, ALT	Non-SVR	All-cause mortality
Innes 2011	HR = **0.19 (.08–.48)**	HR = **0.22 (.09–.58)**	Age, gender, race, genotype, cirrhosis, alcohol-related hospitalization, ever injector, ALT post-treatment	Non-SVR	Liver-related mortality
Backus 2011	GT1: HR = **0.45 (.39–.52)**	GT1: HR = **0.71 (.59–.83)**	Age, gender, treatment duration, cirrhosis, albumin, AST, ALT, creatinine clearance, platelets, sodium, COPD, diabetes, hypertension	Non-SVR	All-cause mortality
	GT2: HR = **0.50 (.38–.65)**	GT2: HR = **0.62 (.46–.88)**			
	GT3: HR = **0.30 (.22–.40)**	GT3: HR = **0.51 (.35–.73)**			
Maruoka 2012	HR = **0.17 (.08–.39)**	HR = **0.17 (.08–.40)**	Age, gender, genotype, fibrosis stage, inflammatory grade, HCV VL, treatment, ALT, platelet, albumin	Untreated	All-cause mortality
Cozen 2013	HR = **0.24 (.10–.58)**	HR = **0.23 (.07–.75)**	Age, race genotype, history of alcohol use, other substance abuse, psychiatric comorbidities, social stability	Untreated	All-cause mortality & LTP
Singal 2013	HR = **0.08 (.02–.34)**	HR = **0.11 (.03–.47)**	Age, gender, race, BMI, genotype, cirrhosis, psychiatric, hypertension, diabetes, albumin, white cell count, platelet count, new referral	Non-SVR	All-cause mortality
Dieperink 2014	HR = **0.31 (.19–.51)**	HR = **0.47 (.26–.85)**	Age, genotype, fibrosis stage, treatment history, diabetes, thrombocytopenia, cardiac disease, depression, psychosis/bipolar, substance use disorder, alcohol use disorder, PTSD, integrated care	Non-SVR	All-cause mortality
Cirrhotic					
Braks 2007	NR	HR = **0.14 (.04–.45)**	Age, sex, genotype, duration of treatment	Non-SVR	Liver-events & mortality
Bruno 2007	HR = **0.13 (.03–.53)**	HR = **0.14 (.04–.59)**	Age, sex, genotype, platelets	Non-SVR	Liver-related mortality
Morgan 2010	NR	HR = **0.17 (.06–.46)**	Age, race, fibrosis stage, AST/ALT ratio, platelets, albumin, alkaline phosphatase, AFP	Non-SVR	All-cause mortality & LTP
Van der Meer 2012	NR	HR = **0.26 (.14–.49)**	Age, gender, BMI, treatment history, diabetes, history of alcohol abuse, fibrosis stage (lab data: platelet count, bilirubin, albumin, AST/ALT ratio, AntiHBc positivity)	Non-SVR	All-cause mortality
		HR = **0.25 (.12–.53)** including lab markers			
Aleman 2013	NR	HR = **0.36 (.18–.68)**	Age, sex, alcohol consumption, diabetes	Non-SVR	All-cause mortality
Kutala 2014	HR = **0.31 (.13–.74)**	HR = **0.35 (.15–.84)**	Age, gender, BMI, genotype, fibrosis stage, HCV VL, alcohol intake, diabetes, hypertension, anti-HBc antigen, AST/ALT ratio, albumin, AFP, bilirubin, creatinine, prothrombin, platelet count	Non-SVR	All-cause mortality & LTP
Coinfected					
Berenguer 2012	HR = **0.25 (.10–.63)**	HR = **0.31 (.12–.83)**	Age, sex, fibrosis stage, cART, HIV VL, nadir CD4, HIV transmission category	Non-SVR	Non-liver related deaths
Mira 2013	HR = **0.23 (.05–.93)**	HR = **0.13 (.02–.93)**	Age, sex, genotype, HCV VL, CDC stage, CD4 count, HIV VL, CTP class, MELD score, liver stiffness	Non-SVR	All-cause mortality
Labarga 2014	NR	HR = **0.12 (.03–.54)**	Age, sex, CD4 count, HIV VL, fibrosis score, IL28B subtype, serum HBsAg	Non-SVR	Liver-events & mortality

HR shown in bold when considered statistically significant.

Abbreviations: AFP, alpha-fetoprotein; ALT, alanine aminotransferase; AntiHBc, hepatitis B core antibody; AST, aspartate aminotransferase; BMI, body mass index; cART, combination antiretroviral therapy; CDC, Centers for Disease Control and Prevention; COPD, chronic obstructive pulmonary disease; CTP, Child Turcotte-Pugh; GT, genotype; HBsAg, hepatitis B surface antigen; HCV, hepatitis C virus; HIV, human immunodeficiency virus; HR, hazard ratio; IL, interleukin; LTP, liver transplantation; MELD, Model End Stage Liver Disease; NR, not reported; NS, non-significant; PTSD, post-traumatic stress disorder; SVR, sustained virologic response; VL, viral load.

**Figure 2.**
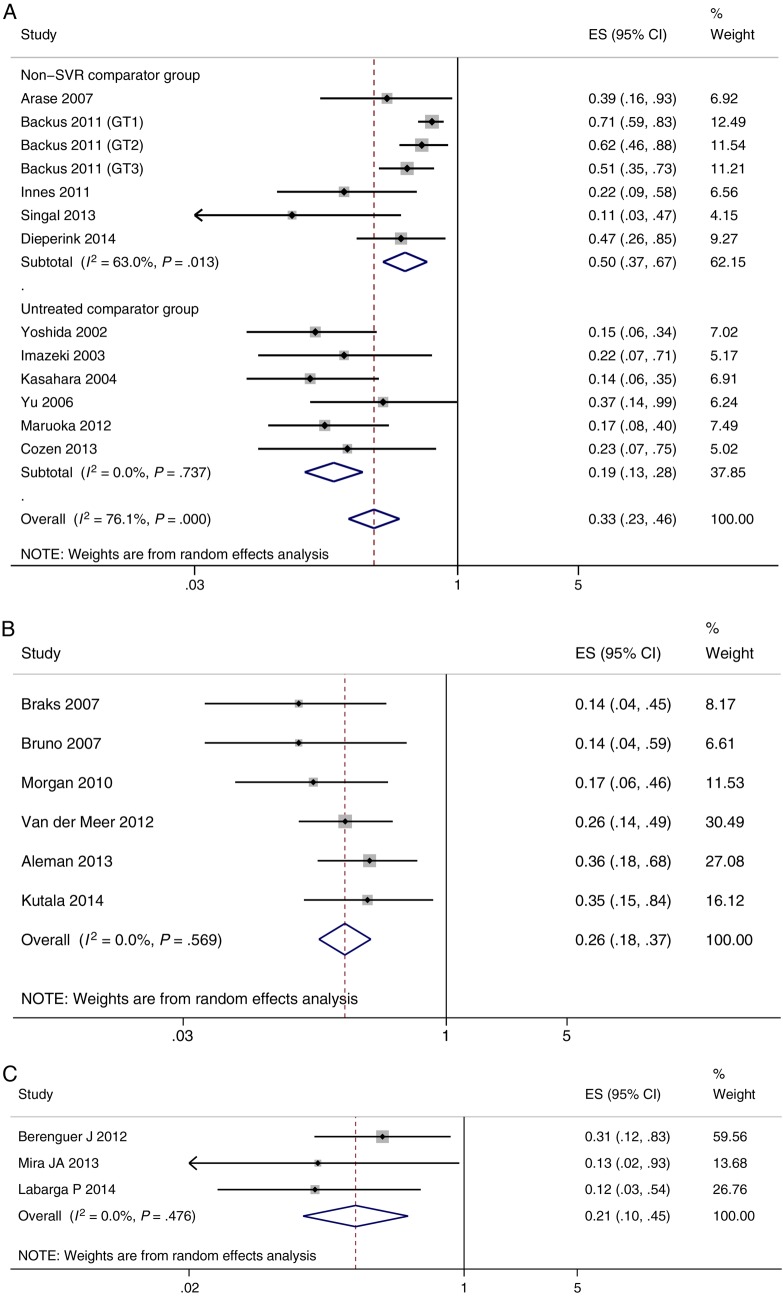
Forest plot of studies and pooled estimates of adjusted hazard ratios of mortality in those achieving sustained virologic response (SVR) vs non-SVR. In (*A*) the general cohort; (*B*) the cirrhotic cohort; and (*C*) the coinfected cohort. Abbreviations: CI, confidence interval; ES, effect size.

The results of the pooled HR analysis are shown in Figure 2*A*–*C*. In all studies SVR-attainment remained a significant predictor of reduced mortality after adjustment for covariates. SVR had the largest protective effect in the coinfected population (HR = 0.21, 95% CI, .10–.45, median follow-up 5.2 years), followed by the cirrhotic population (HR = 0.26, 95% CI, .18–.37, median follow-up 6.8 years), and the general population (HR = 0.33, 95% CI, .23–.46, median follow-up 5.0 years). In the general population considerable heterogeneity between studies was observed (*I*^2^ = 76%, *P* < .0001). As such a subgroup analysis was conducted and it was found that the HR significantly differed when the reference group was an untreated population (HR = 0.19, 95% CI, .13–.28) compared with non-SVR (HR = 0.50, 95% CI, .37–.67; *P* < .0001). This result was confirmed by the funnel plot analysis which showed 2 distinct subgroups of studies (Supplementary Appendix 2). There was no evidence of heterogeneity between studies in both the cirrhotic and coinfected populations (*I*^2^ = 0%), and all studies in these groups compared SVR with non-SVR. Furthermore, based on a funnel plot examination of the cirrhotic and coinfected populations there was no evidence of bias; however, this result should be interpreted with caution due to the small number of studies.

## DISCUSSION

The results of this large meta-analysis investigating the risk of mortality after treatment for chronic HCV indicate that achieving an SVR significantly reduces the risk of death compared with unsuccessful therapy in a variety of populations. After adjustment for potential confounding factors, an SVR was associated with approximately a 50%, 74%, and 79% decreased risk of all-cause mortality compared with not achieving an SVR in the general, cirrhotic, and coinfected populations respectively. The decrease in risk gives rise to a substantially lower 5-year mortality rate in patients achieving SVR compared with nonresponders. This difference was most pronounced in the cirrhotic and coinfected cohorts. Cumulatively, this evidence suggests that there is a significant survival benefit of attaining an SVR, even in patients with cirrhosis and those coinfected with HIV.

Interestingly, the 5-year mortality rate was lowest in patients coinfected with HIV achieving an SVR (1.5%), contradicting existing hypotheses that coinfected patients suffer from higher overall mortality than monoinfected patients [51]. This is likely due to the small number of studies evaluating this population, meaning that differences in absolute reductions in risk are more prominent. Indeed, the risk reduction of death is highest in this population, corroborating evidence that attainment of an SVR can prevent the increased rate of liver-complications associated with HIV coinfection [52].

All-cause mortality was deemed the most appropriate endpoint for a number of reasons. Firstly, there are a number of extra-hepatic complications of chronic HCV that can result in mortality unrelated to liver events [10, 53, 54]. These manifestations of HCV include Type II diabetes mellitus, rheumatic disorders, and cardiac disease [54]. Mortality associated with extra-hepatic disorders may account for why the mortality estimates in the present study are greater than those previously reported [2]. Second, the use of survival as an endpoint is applicable to both high income countries, and low and middle income countries. The aversion of the need for a liver transplant has been used to justify high prices of treatment for HCV; however, for most people infected with HCV, transplantation is not an option.

There are a number of limitations to the current analysis. Above all, there is a concern that the group of patients achieving an SVR systematically differ from patients not achieving an SVR in their baseline characteristics, which may in turn affect outcomes. Patients achieving an SVR tend to be younger, with less severe progression of HCV, and with lower comorbidities, characteristics that could result in lower mortality, regardless of SVR [13, 14, 27, 33]. These potential biases were taken in to consideration by presenting adjusted results, which demonstrate a lower risk of mortality after SVR, independent of other factors. There is some uncertainty over the reliability of these results, as due to differences in the data reported in the literature, the estimates combine different endpoints. Additionally, multivariate analysis may not have been adequate, or in studies where extensive multivariate analyses was carried out, the possibility remains that survival benefit is influenced by additional confounding factors. This criticism would likely be exacerbated when comparing patients achieving SVR with those not treated, given that the present comparator, the non-SVR group, were healthy enough to attempt treatment. The most rigorous way to assess the impact of attaining an SVR on mortality would be to conduct a randomized controlled trial comparing treatment with no treatment [55]. This, however is inappropriate given the related ethical concerns [56, 57]. Furthermore, there was a high risk of bias in relation to the origin of follow-up. A number of studies measured follow-up from treatment initiation, or even earlier than this, rather than the SVR time-point, allowing the accruement of PYFU before SVR-attainment. The impact of this would likely be diminished in the pooled HR analysis given that the origin of follow-up was the same for both arms in each individual study.

The results presented in this analysis are for a 5-year follow-up period due to this being the average follow-up duration. Estimates for a longer timespan would require a greater number of assumptions regarding the relative outcomes between the SVR and non-SVR groups and was thus deemed inappropriate. There is a need for longer-term follow-up to see whether the survival benefit is sustained. Lastly, the current findings are from studies of patients treated with interferon-based treatment, with long-term outcome data currently unavailable for people treated with the more efficacious all-oral therapies.

The results of this meta-analysis suggest that there is a significant survival benefit of achieving an SVR compared with unsuccessful treatment. Moreover, this benefit is held in patients with cirrhosis and those coinfected with HIV. There are no data to support the notion that the value of achieving SVR is influenced by the means used to achieve it. Although the expectation is that patients achieving SVR with interferon free treatment will have at least as much benefit from SVR as seen in historical studies, post-SVR patients cohorts do not yet have sufficient follow-up time to be helpful. Monitoring these outcomes has been built in to a number of registration trial programs, and further data collection over coming years will be important to build on the studies analyzed here.

## Supplementary Data

Supplementary materials are available at *Clinical Infectious Diseases* online (http://cid.oxfordjournals.org). Supplementary materials consist of data provided by the author that are published to benefit the reader. The posted materials are not copyedited. The contents of all supplementary data are the sole responsibility of the authors. Questions or messages regarding errors should be addressed to the author.

Supplementary Data
