# Supplementary material for: Long-Term Treatment Outcomes of Patients Infected With Hepatitis C Virus: A Systematic Review and Meta-analysis of the Survival Benefit of Achieving a Sustained Virological Response
Source: Clin Infect Dis. 2015 May 17;61(5):730–40. doi: 10.1093/cid/civ396 (PMC4530725; doi:10.1093/cid/civ396)
Supplement: Supplementary Data [file supp_61_5_730__index.html]

Long-Term Treatment Outcomes of Patients Infected With Hepatitis C Virus: A Systematic Review and Meta-analysis of the Survival Benefit of Achieving a Sustained Virological Response — Long-Term Treatment Outcomes of Patients Infected With Hepatitis C Virus: A Systematic Review and Meta-analysis of the Survival Benefit of Achieving a Sustained Virological Response — Supplementary Data 

# Long-Term Treatment Outcomes of Patients Infected With Hepatitis C Virus: A Systematic Review and Meta-analysis of the Survival Benefit of Achieving a Sustained Virological Response

## Supplementary Data

Supplementary Data

- Supplementary Data - Docx file
