# Supplementary material for: Long-Term Treatment Outcomes of Patients Infected With Hepatitis C Virus: A Systematic Review and Meta-analysis of the Survival Benefit of Achieving a Sustained Virological Response
Source: Clin Infect Dis. 2015 May 17;61(5):730–40. doi: 10.1093/cid/civ396 (PMC4530725; doi:10.1093/cid/civ396)
Supplement: Supplementary Data [file supp_civ396_civ396supp.docx]

**Supplementary Appendix 1. Assessment of bias summary using QUIPS tool**

|  | **Participation** | **Attrition** | **PF measurement** | **Outcome measurement** | **Study confounding** | **Statistical analysis** |
| --- | --- | --- | --- | --- | --- | --- |
| **General studies** | | | | | | |
| Giannini | Moderate | Low | Moderate | Moderate | Low | N/A |
| Yoshida | Low | Low | High | Low | Low | Moderate |
| Imazeki | Low | Low | High | Moderate | Low | Moderate |
| Veldt | Low | Low | Moderate | Low | Moderate | N/A |
| Kasahara | Low | Low | High | Moderate | Low | Moderate |
| Coverdale | Low | Low | Low | Moderate | Low | Moderate |
| Yu | Low | Moderate | Moderate | Low | Low | Moderate |
| Arase | Moderate | Low | Moderate | Low | Low | Low |
| Backus | Low | Low | Low | Low | Moderate | Low |
| Innes | Low | Low | Low | Low | Low | Moderate |
| Reimer (conference abs) | Low | Low | High | High | High | N/A |
| Di Martino | Low | Low | High | Low | Low | N/A |
| Maruoka | Low | Low | Moderate | Low | Low | Moderate |
| Cozen | Low | Low | High | Low | Low | Moderate |
| Rutter (conference abs) | Moderate | Moderate | Moderate | Moderate | Low | N/A |
| Singal | Low | Low | Moderate | Low | Low | Low |
| Dieperink | Low | Moderate | Moderate | Low | Low | Low |
| **Cirrhotic studies** | | | | | | |
| Kumar | Moderate | Low | High | Low | Low | N/A |
| Braks | Low | Low | Moderate | Low | Low | Moderate |
| Bruno | Low | Moderate | Moderate | Low | Low | Moderate |
| Mallet | Low | Low | Moderate | Low | Low | N/A |
| Morgan | Low | Low | Moderate | Low | Low | Low |
| Iacobellis | Moderate | Low | Low | Low | Low | N/A |
| Van der Meer | Low | Low | Low | Low | Low | Low |
| Aleman | Low | Low | Low | Low | Low | Low |
| K-Kutala | Low | Low | Moderate | Low | Low | Low |
| **Co-infected studies** | | | | | | |
| Limketkai | Low | Low | High | Moderate | Low | N/A |
| Berenguer | Low | Low | Moderate | Low | Low | Moderate |
| P-Gonzalez | Moderate | Low | Low | Low | Low | N/A |
| Mira | Moderate | Low | Low | Low | Low | Low |
| Labarga | Moderate | Low | Low | Low | Low | Moderate |

**Supplementary Appendix 2. Funnels plots to examine bias for adjusted hazard ratios of mortality in those achieving SVR versus non-SVR. In (a) the general cohort; (b) the cirrhotic cohort; and (c) the co-infected cohort.**


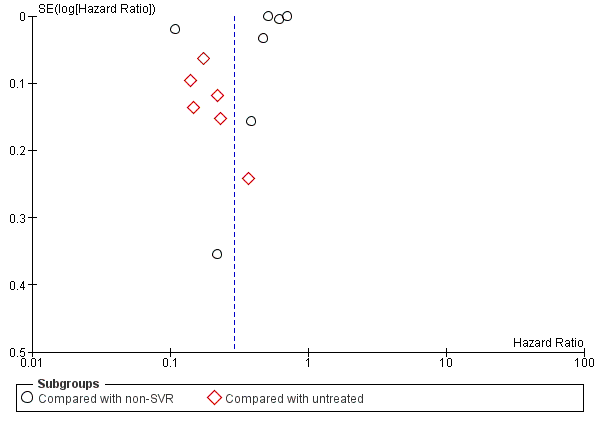


A)


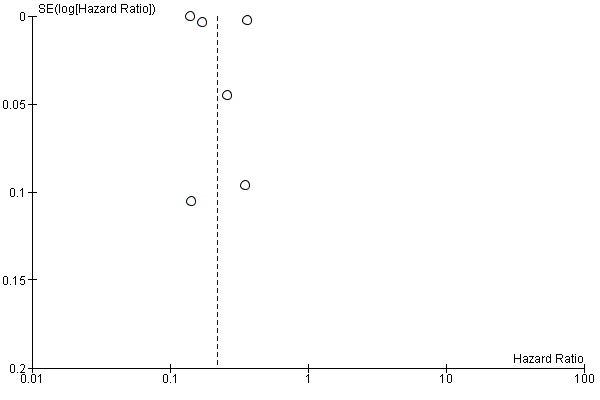


B)


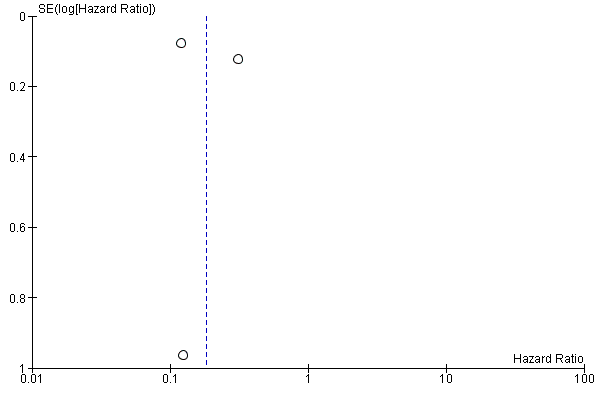


C)
